# Automatic segmentation of the gross target volume in radiotherapy for lung cancer using transresSEUnet 2.5D Network

**DOI:** 10.1186/s12967-022-03732-w

**Published:** 2022-11-12

**Authors:** Hui Xie, Zijie Chen, Jincheng Deng, Jianfang Zhang, Hanping Duan, Qing Li

**Affiliations:** 1 Department of Radiation Oncology, Affiliated Hospital (Clinical College) of Xiangnan University, Chenzhou, 423000 People’s Republic of China; 2 Key Laboratory of Medical Imaging and Artifical Intelligence of Hunan Province, Chenzhou, 423000 People’s Republic of China; 3 Shenying Medical Technology (Shenzhen) Co., Ltd. Shenzhen, Shenzhen, 518057 China; 4 Department of Physical Examination, Beihu Centers for Disease Control and Prevention, Chenzhou, 423000 People’s Republic of China; 5 Department of Nuclear Medicine, Affiliated Hospital (Clinical College) of Xiangnan University, Chenzhou, 423000 Hunan Province People’s Republic of China; 6 School of Medical Imaging, Laboratory Science and Rehabilitation, Xiangnan University, Chenzhou, 423000 Hunan Province People’s Republic of China

**Keywords:** Lung cancer, GTV, Medical image segmentation, Radiotherapy, Residual connection, Dual attention mechanism

## Abstract

**Objective:**

This paper intends to propose a method of using TransResSEUnet2.5D network for accurate automatic segmentation of the Gross Target Volume (GTV) in Radiotherapy for lung cancer.

**Methods:**

A total of 11,370 computed tomograms (CT), deriving from 137 cases, of lung cancer patients under radiotherapy developed by radiotherapists were used as the training set; 1642 CT images in 20 cases were used as the validation set, and 1685 CT images in 20 cases were used as the test set. The proposed network was tuned and trained to obtain the best segmentation model and its performance was measured by the Dice Similarity Coefficient (DSC) and with 95% Hausdorff distance (HD95). Lastly, as to demonstrate the accuracy of the automatic segmentation of the network proposed in this study, all possible mirrors of the input images were put into Unet2D, Unet2.5D, Unet3D, ResSEUnet3D, ResSEUnet2.5D, and TransResUnet2.5D, and their respective segmentation performances were compared and assessed.

**Results:**

The segmentation results of the test set showed that TransResSEUnet2.5D performed the best in the DSC (84.08 ± 0.04) %, HD95 (8.11 ± 3.43) mm and time (6.50 ± 1.31) s metrics compared to the other three networks.

**Conclusions:**

The TransResSEUnet 2.5D proposed in this study can automatically segment the GTV of radiotherapy for lung cancer patients with more accuracy.

## Innovation

1. The proposed 2.5D architecture for residual connection uses 2D convolutional layers to extract 2D edge feature information of targets in CT images and accurately restore edge details in segmentation results, and 3D convolutional layers to extract abstract semantic features by exploiting interlayer information in CT images.
2. We proposed the adoption of Res-Dual-Attention Module, which uses the dual attention mechanism of channel attention brought by SE Block and global attention brought by Transformer, and combines two different operators, Convolution and Transformer, to extract local features and global features simultaneously.

## Introduction

In February 2022, the National Center of Cancer (China) released the latest national cancer statistics [1]: lung cancer is the number one malignant tumor in China in terms of incidence and the number one cause of cancer deaths. According to the International Agency for Research on Cancer (IARC) of the World Health Organization (WHO) [2], the number of new lung cancer cases in 2021 was 2.2 million, ranking second only to breast cancer with 2.26 million cases; and there were 1.8 million lung cancer deaths, out of the 9.96 million cancer deaths worldwide, far exceeding the death rate of other cancers and making lung cancer the mostly deadly cancer type. It is clear that lung cancer poses a great threat to human health.

Radiation therapy is one of the main treatments for lung cancer, and about 60%-70% of lung cancer patients need to receive radiation therapy [3]. In recent years, with the rapid development of medical imaging technology and computer technology, we have entered the era of image-guided high-precision radiotherapy in tumor radiotherapy. Precision radiotherapy is initially based on the manual outlining of the radiotherapy target area and the endangered organs by the medical professionals [4]. The normal delineation of target areas and organs-at-risk (OARs) is a key step in tumor radiotherapy planning. In order to reduce the complications of radiotherapy and the risk of secondary malignant tumor caused by radiation, it is necessary to accurately delineate the target area and OARs. Even though there are unified principles and consensus for reference, the manual outlining of radiotherapy targets is still largely based on the experience of the practitioner [4]. This method is highly variable and time-consuming, which has an impact on the efficacy of radiation therapy. Artificial intelligence is increasingly used in the medical field [5], and the use of artificial intelligence techniques can provide optimized and effective decisions with minimal error, offering unparalleled advantages in improving the efficiency and consistency of target outlining in radiotherapy. Convolutional neural network (CNN) is a type of deep learning and has better results in medical image segmentation because CNN is insensitive to image noise, blur, and contrast [6] and is currently one of the most successful algorithms to achieve image segmentation. In the field of tumor radiology, a trained CNN model, accelerated by a graphics processing unit (GPU), can achieve the task of fast segmentation of the Gross Target Volume (GTV) as well as normal tissues and organs. Rhee et al*.* [7] used CNN for automatic segmentation of the clinical target volume (CTV) of pelvic tumors on CT localized in radiotherapy and achieved a DSC of 0.86. Men et al*.* [8] used an end-to-end Deep Deconvolutional Neural Network to segment the primary lesion of nasopharyngeal carcinoma with a DSC of 0.809. Wang et al*.* [9] proposed a new patient-specific adaptive convolutional neural network (A-net), which used the weekly MRI images and the segmentation of the GTV to train this network, with a DSC of 0.82 ± 0.10. Zhang et al*.* [10] introduced a modified ResNet to segment the GTV of non-small cell lung cancer patients on the CT images, with the average DSC level of 0.73. Although deep learning based automatic segmentation techniques have rapidly applied in delineating the OARs and GTV in lung cancer radiotherapy, when it comes to automatic segmentation of the GTV in radiotherapy planning for lung cancer, the studies are still not much or deep enough, and the segmentation performance is not very well. Therefore, there is an urgent need for a method to automatically segment the GTV of lung cancer in the field of radiotherapy to improve the efficiency and accuracy of GTV outlining.

In this paper, we propose that a TransResSEUnet2.5D network can perform accurate segmentation of GTV in radiotherapy for lung cancer, and greatly save the time for segmentation.

## Materials and methods

### TransResSEUnet2.5D network

#### Network architecture design

**Fig. 1:**
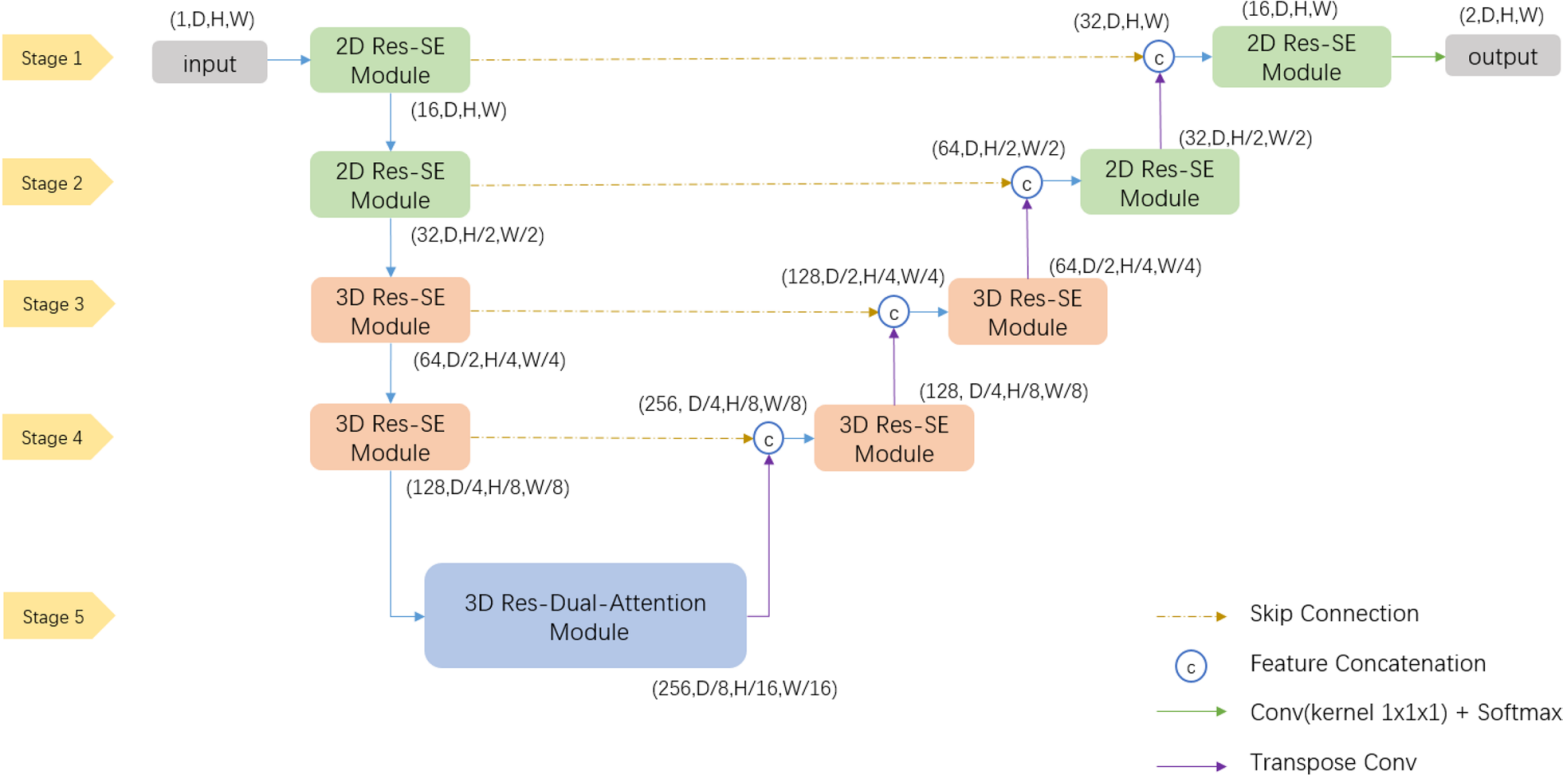
Overall network architecture diagram

The TransResSEUnet 2.5D network proposed in this study is a 3D CNN network based on an Encoder-Decoder architecture (3D Unet [11]), as shown in Fig. 1. The encoder part is used to extract edge features and semantic features, and the decoder part performs Feature Concatenate by fusing low-level features with high-level features through Skip Connection, and up-samples high-level features using Transpose Convolution to gradually recover to the original resolution of the input image. The probability maps of the background and target classes are calculated as the output of the network using a convolutional layer with a 1 × 1×1 kernel and a Softmax layer.

#### Res-SE module

**Fig. 2:**
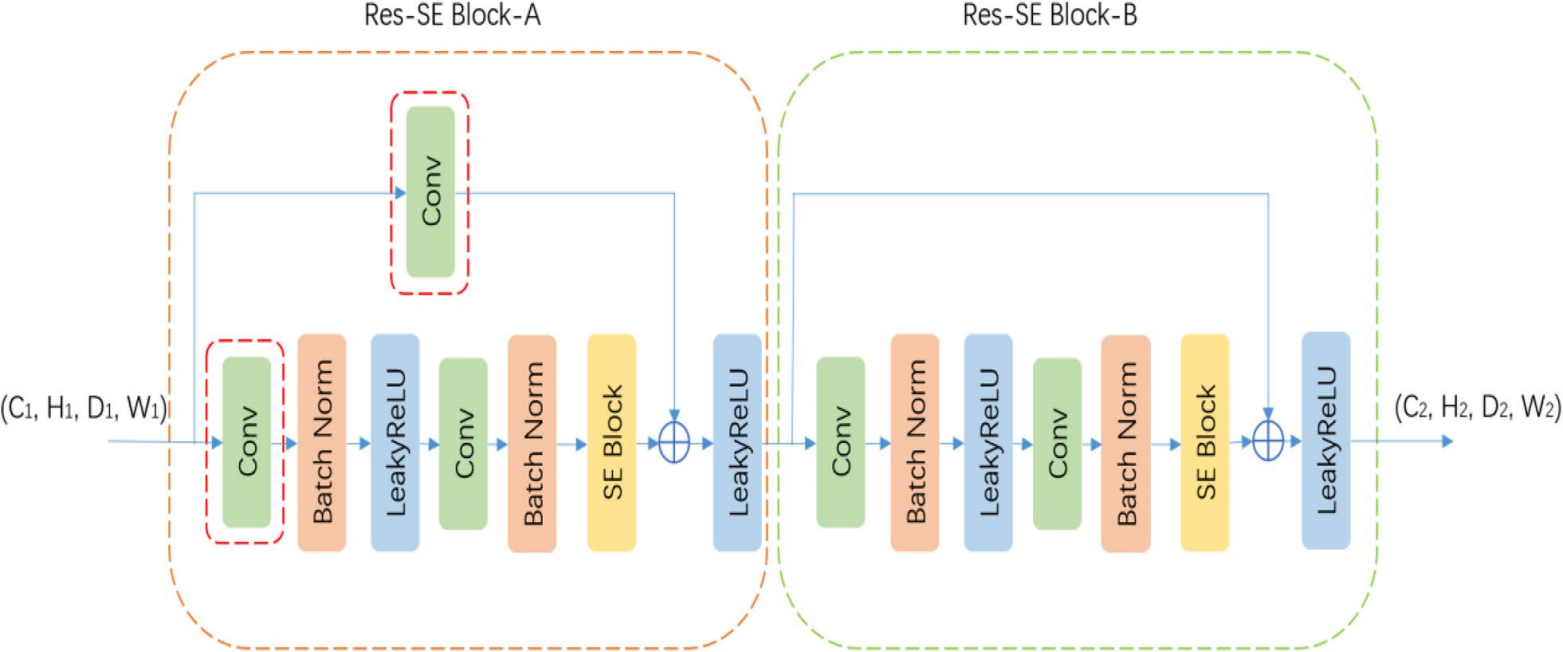
Res-SE Module

The TransResSEUnet2.5D uses the Res-SE Module as the basic unit for feature extraction. As shown in Fig. 2, the Res-SE Module consists of two parts, Res-SE Block-A and Res-SE Block-B, where the use of residual connection [12] effectively alleviates the problem of gradient dispersion. The difference between the two is that, Res-SE Block-A serves to reduce the spatial resolution and the number of channels of the feature map by modifying the convolution step and the number of output channels of the convolution layer in the red dashed box, while Res-SE Block-B does not have any effect on the spatial resolution and the number of channels of the feature map and is only used to extract features.

Since the activation function ReLU [13] (Linear rectification function) is constant to 0 in the negative region, when the learning rate is too high and the parameters of the model are adjusted too much, it may cause the output of a large number of neurons to be set to 0. If all the neurons in a certain hidden layer are set to 0, it will cause an interruption in training, and once the neurons are set to 0, they will not recover and proceed to a permanent death, as the neuronal parameters will not be updated and the Leaky ReLU [14] (Leaky linear rectification function) gives a non-zero slope to all negative values; therefore, Leaky ReLU is used as the activation function and the slope is set to 0.01.

Batch Normalization [15] (BN) layer is introduced between each convolutional layer and the activation function, and the BN layer is used to calculate the mean and variance of the output of the previous convolutional layer to transform the data into a stable distribution with the mean of 0 and deviation of 1. By doing so, we can effectively prevent gradient explosion or dispersion, reduce the dependence on the initialization parameters of the network, allow the use of a larger learning rate in training, and regularize the network to a certain extent, thus the convergence speed of the network will be accelerated, and the accuracy of target recognition will be enhanced.

**Fig. 3:**
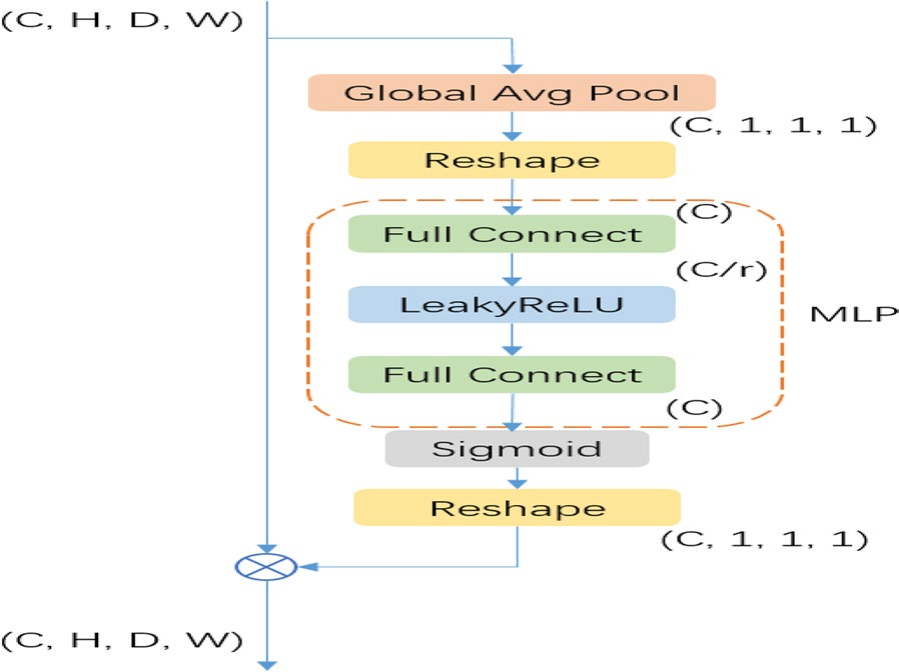
SE Block

The squeeze and excitation module (SE Block) is introduced after the second BN layer in each Res-SE Block. The SE Block was first used for target classification [16] and is now increasingly used in segmentation tasks. As shown in Fig. 3, the first step of the SE Block uses the Global Average Pool (GAP) layer to compress the spatial resolution of the feature map to a size of 1 × 1×1 and collapse the feature map into a one-dimensional vector. The second step uses the Multilayer Perceptron [17] (MLP, Multilayer Perceptron), wherein the number of channels in the implicit layer is reduced to one rth of the input channels; the reduction of the number of channels leads to information loss and facilitates the removal of redundant information, and the decay factor r, which controls the degree of information loss, is a hyperparameter. Hu et al*.* [16] have experimented with different values of 2, 4, 8, 16, etc., and found that the classification accuracy is highest when r = 16, and since the minimum number of channels in the convolutional layer in our study is a 1/4 of that of the Hu’s study [16], so r = 16/4 = 4 is taken. The third step uses Sigmoid to normalize the feature values to between 0 and 1 and restore them to their original dimensions to obtain the weights of each feature channel in the input feature map. Finally, the weights of the feature channels are multiplied with the input feature map to play the role of enhancing useful features and suppressing useless features, so that subsequent layers learn from them. The weights of the feature channels are multiplied with the input feature map to enhance the useful features and suppress the useless features, so that the subsequent layers can learn new features with more discriminative power for target segmentation and finally achieve a more accurate target segmentation.

#### 2.5D architecture

**Fig. 4:**
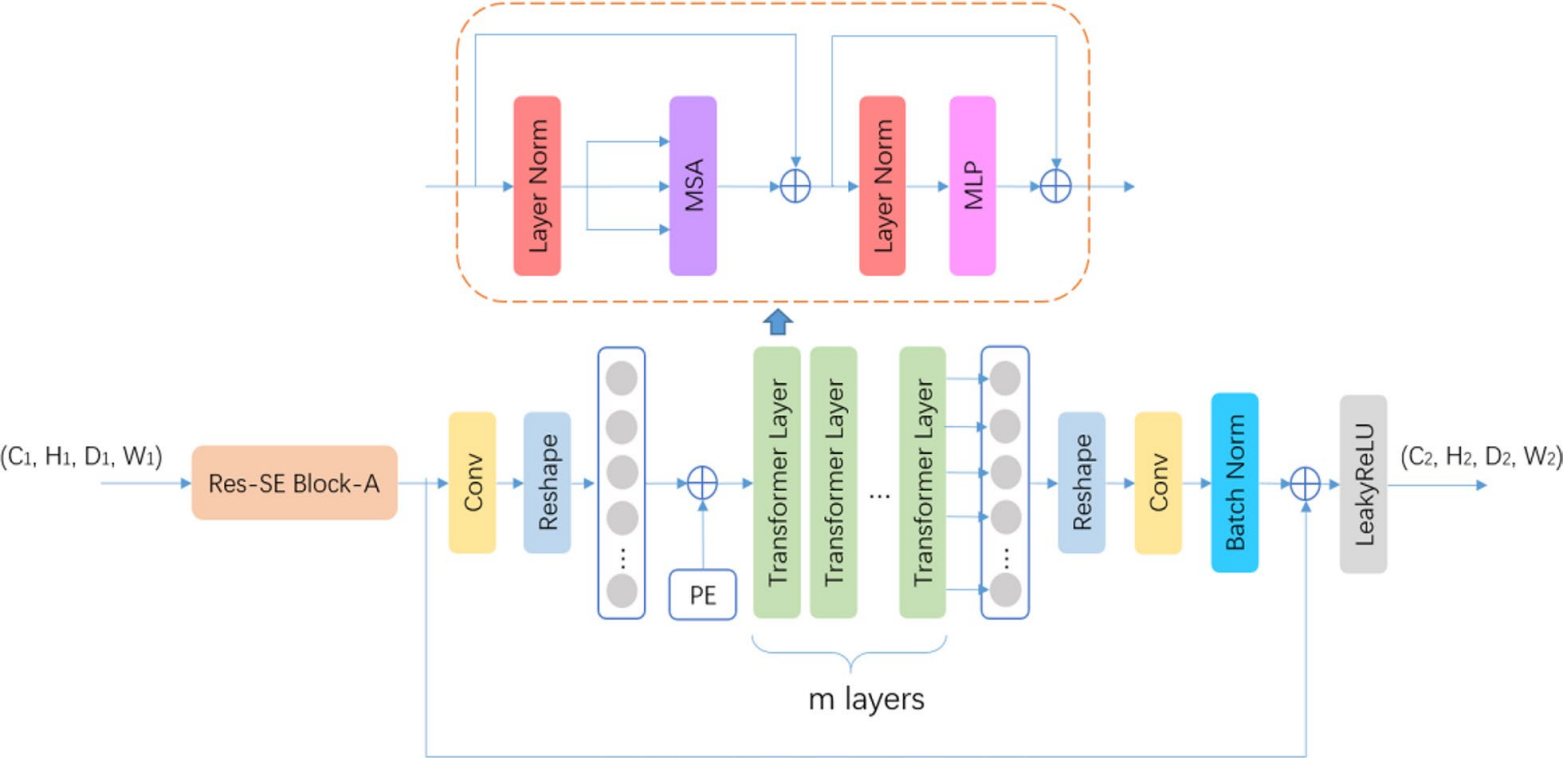
Res-Dual-Attention Module

Chen et al*.* [18] found that the pixel spacing of anisotropic CT causes the edges of segmented targets to be sharper in two dimensions and rougher and fuzzier in three dimensions, leading to the difficulty for the network to learn the edge detail features of the target and resulting in the outcome that the final segmented target contours are less close to the edges of the segmented targets. In deep neural networks, the lower layer features carry more edge information, while the higher layer features carry more abstract semantic information. The 2D convolutional layers are better at extracting the edge features of the segmentation target, while the 3D operation is better at extracting the semantic features of the segmentation target in three dimensions, so Chen [18] proposed a 2.5D architecture, in which 2D convolutional layers are used in the upper part of the network to extract the 2D edge feature information of the target in the CT image and accurately restore the edge details in the segmentation result; and 3D Convolutional layers are used in the lower part to extract abstract semantic features from the interlayer information in the CT images. The layer thickness of CT in the dataset of our study is about 5 times the pixel spacing in x and y directions, which is 3 times of that of the study of Chen [18], so it is more suitable to use 2.5D architecture [18] (See Fig. 4).

As shown in Fig. 1, the Res-SE Module in the encoder and decoder parts of Stage 1 and Stage 2 of the network is defined as a 2D module, i.e., the convolutional kernel size and convolutional step size in all convolutional layers are set to 1 in D dimension to focus on learning the 2D edge features of the segmentation target; and then the 3D Res-SE Module in the encoder and decoder parts of Stage 3 and Stage 4 is used to learn the semantic features of the segmentation target in 3D space; and lastly, the Res-Dual-Attention Module is used in Stage 5 to extract more discriminative features using a dual-attention mechanism.

#### Res-dual-attention module

Local features are compact vector representations of local neighborhood, while global features include contour representations, shape descriptors, and object representations over long distances, etc. Both are extremely important for image segmentation tasks. In CNN, convolutional operations are good at extracting local features, but still have limitations in capturing global feature representations. In contrast, Transformer [19], designed for sequence-to-sequence prediction, has an innate global self-attention (GSM) mechanism, which is not only powerful in modeling global context, but also shows excellent transferability to downstream tasks under large-scale pre-training. It has been successfully used in machine translation and natural language processing [20, 21]. In recent years, the attempts at various image recognition tasks with Transformer have also met or even exceeded state-of-the-art performance [22–24].

However, because Transformer treats the input as a 1D sequence and focuses on modeling the global context at all stages, it leads to low-resolution features that lack detailed localization information. As such information cannot be recovered efficiently by directly up-sampling to full resolution, coarser segmentation results are consequently produced. Therefore, our study proposes the adoption of the Res-Dual-Attention Module, which utilizes the dual attention mechanism of channel attention brought by SE Block and global attention brought by Transformer and combines two different operators of convolution and Transformer to extract local features and global features simultaneously.

Res-Dual-Attention Module is divided into two serially connected sub-modules. The first step uses Res-SE Block-A to downsample the input feature map and increase the feature channel to C_2_, and the second step uses Res-Trans Block to extract global features. Res-Trans Block is divided into four steps.

Image Serialization: Res-Trans Block first uses a convolutional layer for linear mapping, increasing the feature channels to C_3_ = 512, and then tokenizes the feature map by collapsing the D_2_, H_2_, and W_2_ dimensions into one dimension to form a feature map f of C_3_ x P (P = D_2_ x H_2_ x W_2_), which can be treated as P tokens whose individual lexical element is encoded with the length C_3_.

Position Embedding: To encode the spatial location information of each lexical element, this study uses a learnable position embedding module and preserves the spatial location information by adding it directly to the feature map f.

1 $${\text{z}}_{0} = F + PE$$

Transformer Layers: Res-Trans Block uses M Transformer layers, where each Transformer layer consists of Multi-Head Self-Attention (MSA) and Multi-Layer Perceptron (MLP) (Eqs. 2–3), so the output of the Transformer in the mth layer can be expressed as the following equation

2 $${\text{z}}_{m}^{ * } = MSA\left( {LN\left( {z_{m - 1} } \right)} \right) + z_{m - 1}$$

3 $${\text{z}}_{m} = MLP\left( {LN\left( {{\text{z}}_{m}^{ * } } \right)} \right) + z_{m}^{*}$$

LN refers to Layer Normalization (Layer Normalization [25]),${\text{z}}_{{\text{m}}}$ denoting the output of the Transformer at the mth layer; MSA contains H self-attentive modules; and MLP is a three-layer perceptron with an implicit layer h. By experiments, the segmentation is best when M = 4, H = 8, and h = 4096.

Image deserialization: Since the input dimension of the subsequent decoder part is a 4-dimensional tensor, the feature sequence output from Transformer Layers is expanded into a 4-dimensional tensor, and a convolution layer is used to reduce the number of feature channels to 256 and add up with the input feature maps of sub-modules to form a residual structure, which is beneficial to the training of Transformer Layers with a higher number of parameters.

### Experiment and analysis

#### Construction of the data set

The dataset for this study was obtained from the localized CT images of 127 lung cancer patients from the Radiotherapy Center of Xiangnan College Hospital, and the CT images of 50 lung cancer patients, which were provided by the Automatic Structure Segmentation for Radiotherapy Planning Challenge 2019 (MICCAI StructSeg2019) in the dataset titled *Gross Target Volume segmentation of lung cancer* [26]. The applied images in this study were collected by a large-aperture slice spiral CT simulator (Phillips Medical System, Brilliance CT Big Bore, OH, USA) of the Affiliated Hospital of Xiangnan University, according to a standardized CT acquisition protocol: tube voltage 120 V, tube current 250 mAs, layer thickness 5 mm, layer spacing 5 mm, resolution Standard, matrix 512 × 512. By parsing the DICOM file, the grayscale values of the original image CT were mapped to the range of 0–255, and the window width of 400 and window position of 40 were adjusted to change the contrast and brightness of the images. The GTV contour of lung cancer, which was manually outlined by the oncology radiologist, was mapped onto the original image with a resolution of 512 × 512, and the grayscale values were filled according to the key values of GTV to generate a mask map as the label for training. All datasets were manually segmented by two radiotherapists, who followed the guidelines for lung cancer treatment provided by the Chinese Society of Clinical Oncology (CSCO, CSCO) [27] and the National Comprehensive Cancer Network (NCCN) network [28], and then confirmed by two radiotherapists with the title of associate chief physician or higher.

The training set includes a total of 11,370 CT images, which were taken from 87 patients from the hospital where the authors of this study work, and 50 patients whose information became available through the miccai StructSeg2019. The validation set is 1642 CT images of 20 patients from our hospital. The test set consists of 1685 CT images of 20 patients. After data cleaning and enhancement, they were transported to TransResSEUnet2.5D for training.

#### Implementation details

The input image size is 32 × 256×256, batch size is 4, the optimization method is Adam, the initial learning rate is 1e-3, the weight decay factor is 1e-4, and the polynomial learning rate decay strategy with a power of 0.9 is used to train a total of 500 epochs. Software and systems used in the study include Ubuntu Server 20.04, CUDA11.1, cuDNN8.4, and the PyTorch deep learning framework of v1.10.0. All training was done on two RTX3090 model GPUs. To prevent overfitting, data enhancement techniques such as random cropping, random panning, random rotation, random scaling, random Gaussian noise, and random mirroring were used to expand the size of the training set. The training loss function uses the average Dice loss function [29] and the cross-entropy loss function, and the loss values of both are summed as the total loss value.

#### Experimental design

To demonstrate the effectiveness of TransResSEUnet2.5D and the improvement of each part, four experiments were done separately. (1) Unet3D uses the classical 3D Unet architecture with two 3DConv + BN + LeakyReLU Modules for the encoder and decoder parts of each stage, respectively; (2) ResSEUnet3D replaces 3DConv + BN + LeakyReLU with 3D Res-SE Module; (3) ResSEUnet2.5D replaces the 3D Res-SE Module with 2D Res-SE Module in the first two stages; (4) TransResSEUnet2.5D is the network proposed in this paper.

#### Splitting accuracy evaluation

In this study, we calculated Dice for the entire image sequence of each patient, three-dimensionally, and the Dice Similarity Coefficient (DSC) [29] and 95% Hausdorff Distance (HD95) [30] were used to evaluate the automatic segmentation results of the test set.

4 $$DSC = \frac{{2|V_{A} = V_{B} |}}{{|V_{A} | + |V_{B} |}}$$

$V_{A}$: segmentation results provided by radiotherapists;$V_{B}$: segmentation results obtained from the network.

5 $$HD95 = \frac{1}{2}\left[ {K_{95} \min {\text{d}}\left( {z,S_{A} } \right) + K_{{{95}}} \left( {\min {\text{d}}\left( {z,S_{B} } \right)} \right)} \right]$$

$S_{A}$: surface of the segmentation results provided by radiotherapists; $S_{B}$: surface of the segmentation results obtained from the network; $d(z,S_{A} )$ is the shortest distance from the surface voxel *Z* of the segmentation results obtained from the network to the surface of the segmentation results provided by radiotherapists.

$S_{A}$;$d(z,S_{B} )$ is the shortest distance from the surface voxel *Z* of the segmentation result provided by the radiotherapists to the surface of the segmentation result obtained from the network $S_{B}$;$K_{95}$ indicates 95%.

### Statistical analysis

Statistical analysis was performed using SPSS 21.0 statistical software. The measurement data were tested for normal distribution using the Kolmogorov–Smirnov test and expressed as the mean ± standard ($\overline{x} \pm s$). One-way analysis of variance (ANOVA) with Dunnett’s multiple comparisons test was used for multiple comparisons of normal distribution between groups. Dunnett’s T3 was used for multiple comparisons of non-normality distribution between groups. The significance alpha level was set at 0.05, and *P* < 0.05 indicated that the difference was statistically significant.

## Results

In this study, a total of 11,370 CT images of radiotherapy for lung cancer patients and the GTVs manually outlined by radiotherapists were used as the training set to train our newly designed TransResSEUnet2.5D network for automatic image segmentation. The consistency of the tuned and trained TransResSEUnet2.5D network model was verified by using 20 sets of 1642 CT images and GTVs outlined by radiotherapists as the validation set, and the accuracy of the network for automatic segmentation was determined by DSC and HD95 analysis. Finally, the validity and accuracy of the TransResSEUnet2.5D network model were tested with a test set consisting of 20 sets of 1685 CT images, and the automatic segmentation performance of the network model for radiotherapy localized images was assessed thoroughly.

### TransResSEUnet2.5D network training

**Fig. 5:**
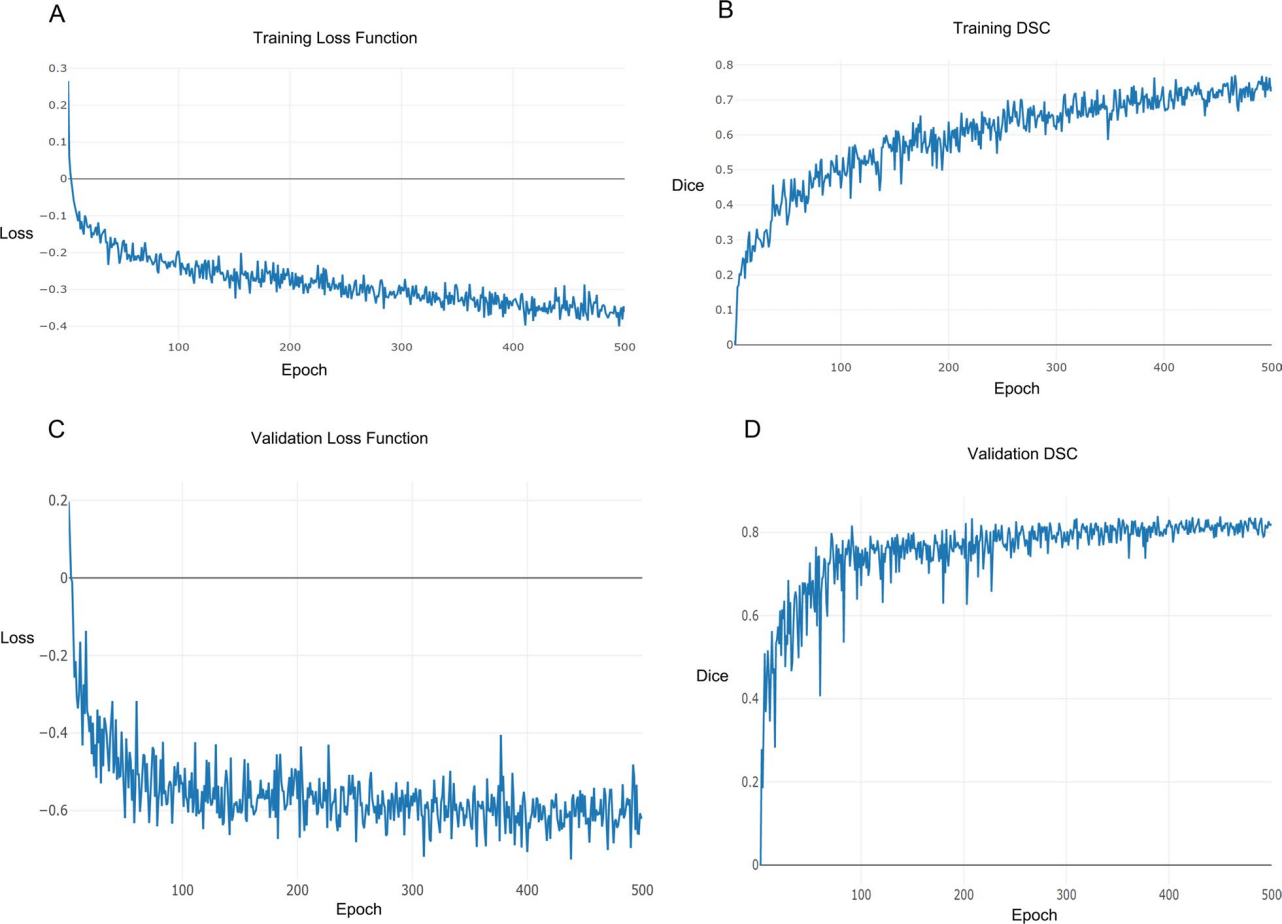
TransResSEUnet2.5D network training and validating. Training loss function curve for each epoch (**A**), and training DSC curve for each epoch (**B**), validation loss function curve for each epoch (**C**), and validation DSC curve for each epoch (**D**)

During the segmentation training of the network, the CT images of the validation set are input to the current model every training cycle (epoch) to get the predicted segmented images, and the average value of DSC is counted based on the real segmented images. If this DSC value is larger than the DSC values of all previous training cycles, the training model is saved and recorded as the best training model. When the training is finished, the CT images of the test set are input to the best training model to get the predicted segmented images, and the average values of DSC and HD95 are counted based on the real segmented images. Figure 5A, B demonstrate the convergence of the loss and DSC values of the training set as the iteration period increases during the training of the network, and Fig. 5C, D show the convergence of the loss and DSC values of the validation set as the iteration period increases during the training of the network model. By comparing the training and validation curves, it is easy to observe that the training DSC and loss are worse than the validation DSC and loss, which might be because the training is a random crop of 32 × 256×256 size image blocks for 3D images, the validation is to crop 32 × 256×256 image blocks in the center of the GTV, the difficulty of these two is not the same, and the image block during the validation is easier to segment out of the GTV, so that the DSC is larger and the loss is smaller. The TransResSEUnet2.5D network constructed in this study has converged after 500 epochs during the training process. The model also has a relatively stable generalization ability after 300 epochs.

### Network architecture comparison

**Fig. 6:**
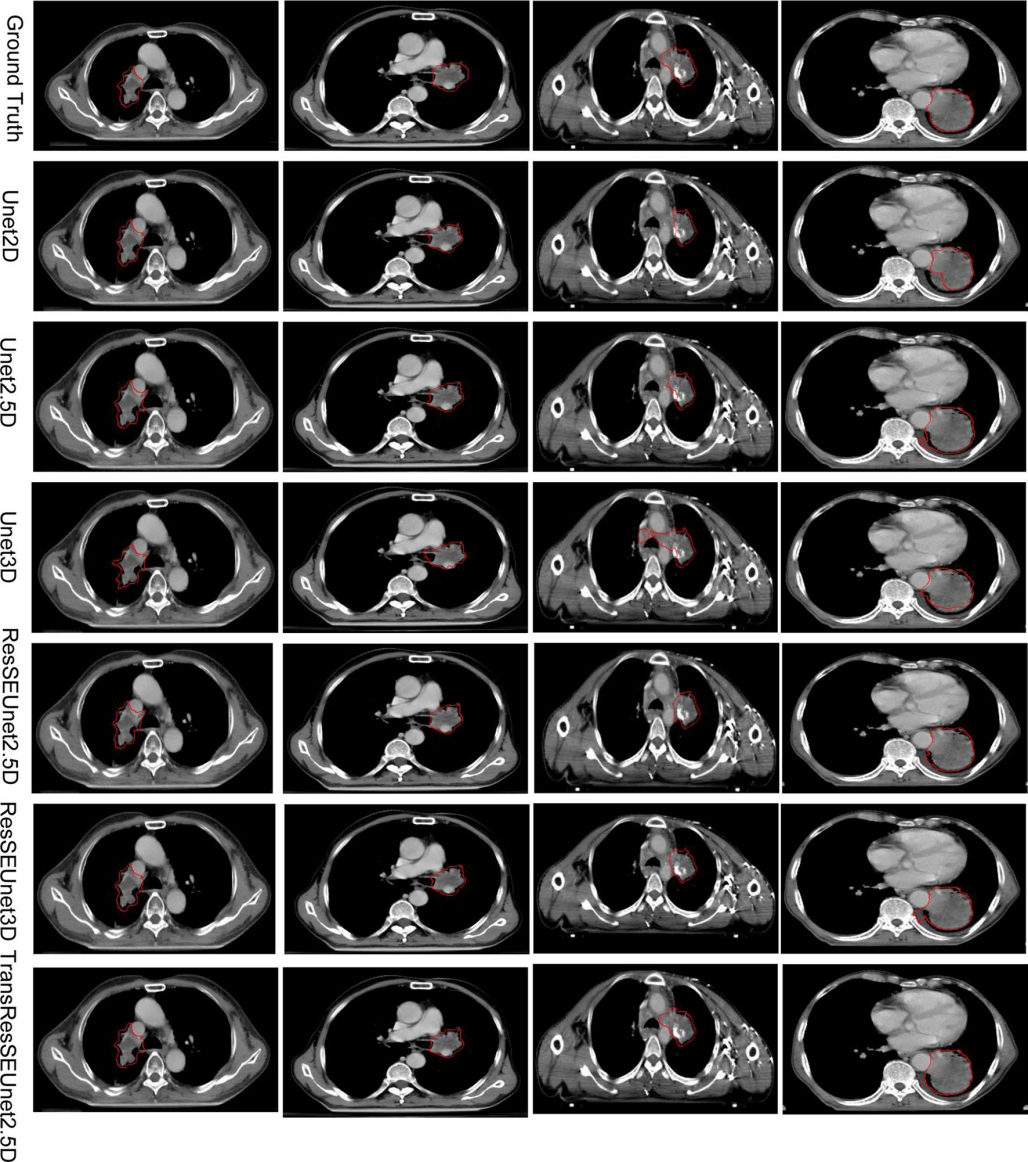
Results of different network structures for the GTV segmentation of radiotherapy for lung cancer

Figure 6 shows the results of automatic segmentation of the GTV of radiotherapy for lung cancer patients using UNet2D, UNet2.5D, Unet3D, ResSEUnet3D, ResSEUnet2.5D, and TransResUnet2.5D networks, respectively. Compared to the other network models, the segmentation results presented in this study are closer to the manual outline results of the radiotherapists. It can also be seen from Fig. 6 that the segmentation results of the remaining five automatic segmentation networks showed false-positive discrete regions and under-segmentation. The TransResSEUnet 2.5D network, on the other hand, has constraints on the strong shape of the segmentation, the segmentation results are more complete, and the problems of false-positive region and under-segmentation are effectively controlled. At the same time, the GTV edges manually outlined by the radiotherapists have jagged noise because the radiotherapists cannot do pixel-level adjustment when outlining, while the GTV regions segmented by the TransResSEUnet2.5D model have smoother boundaries and better fit the actual GTV state because they are detected at the pixel level.

From Table 1 the statistical results of the six network models for automatic segmentation of the GTV in radiotherapy for lung cancer patients are more clearly shown, including the mean DSC, mean HD95 and mean time of each model. In comparison to the other network models, the TransResSEUnet2.5D network model proposed in this paper has the highest DSC of (84.08 ± 0.04) % and the lowest HD95 of (8.11 ± 3.43) mm, indicating that the TransResSEUnet2.5D network model that we proposed can obtain superior automatic segmentation results than the other models. However, in terms of statistical multiple comparisons, compared with the other five models, TransResSEUnet 2.5D does not show differences in DSC and HD95. Table 1 also implied that TransResSEUnet 2.5D presented in this study takes the longest time to automatically segment GTV in lung cancer patients (6.50 ± 1.31) S. Compared with Unet 2.5D (3.69 ± 0.72), Unet3D (3.51 ± 0.58), and ResSEUnet 2.5D (5.52 ± 1.10), TransResSEUnet 2.5D shows statistical difference in the average segmenting time (P < 0.05). This might be due to the complexity of the model.

**Table 1:** Multiple comparison of experimental result indicators of 6 networks ($$\begin{gathered} \overline{x} \pm s \hfill \\ \hfill \\ \end{gathered}$$)

Metric	Model($\overline{X} \pm S$)		P	95% Confidence interval	
				Lower bound	Upper bound
DSC (%)	TransResSEUnet2.5D (84.08 ± 0.04)	Unet2D (77.07 ± 0.09)	0.090	− 0.006	0.146
		Unet2.5D (81.51 ± 0.06)	0.910	− 0.032	0.083
		Unet3D (74.53 ± 0.17)	0.358	− 0.419	0.233
		ResSEUnet3D (80.56 ± 0.06)	1.000	− 0.042	0.064
		ResSEUnet2.5D (82.97 ± 0.06)	0.456	− 0.018	0.088
HD95 (mm)	TransResSEUnet2.5D (8.11 ± 3.43)	Unet2D (15.25 ± 9.04)	0.050	− 14.278	0.002
		Unet2.5D (9.69 ± 6.30)	0.996	− 6.797	3.625
		Unet3D (13.91 ± 13.47)	0.662	− 16.179	4.594
		ResSEUnet3D (11.14 ± 5.43)	1.000	− 4.932	3.550
		ResSEUnet2.5D (8.80 ± 4.81)	0.486	− 7.658	1.612
Average prediction time of single series (s)	TransResSEUnet2.5D (6.50 ± 1.31)	Unet2D (6.30 ± 2.29)	0.944	− 1.377	0.807
		Unet2.5D (3.69 ± 0.72)	0.000	1.644	3.889
		Unet3D (3.51 ± 0.58)	0.000	1.853	4.035
		ResSEUnet3D (4.69 ± 0.96)	0.301	− 0.318	2.191
		ResSEUnet2.5D (5.52 ± 1.10)	0.001	0.557	2.9574

## Discussion

During the planning of radiotherapy, radiotherapists need to outline the GTV on CT images layer by layer. The quality of GTV outlining determines 60% of the overall radiotherapy effectiveness [31]. Manual outlining by physicians is prone to introduce subjective errors and poor traceability. Therefore, a rapid and automated GTV outlining method is important for improving the overall efficiency and performance stability of radiotherapy in clinical practice. Currently, researchers have used machine learning methods to achieve automatic GTV segmentation during radiation treatment for nasopharyngeal carcinoma [32, 33], brain tumors [34], and breast cancer [35]. Li et al*.* [32] used a U-net network to automatically segment the primary lesion of nasopharyngeal carcinoma with a DSC of 0.659; Cardenase et al*.* [33] used a two-channel 3D convolutional neural network for automatic segmentation of the GTV of nasopharyngeal carcinoma with a DSC accuracy of 0.75, and some later studies on automatic segmentation in nasopharyngeal carcinoma achieved a DSC accuracy of up to 0.835 [36].Yang et al*.* [34] proposed a DCU-Net model with a DSC of 0.91 for automatic segmentation of intracranial tumors. In another study using DD- Res Net network for postoperative breast cancer also achieved a DSC of 0.91 for automatic segmentation of CTV [35]. In lung cancer, some progress has been made. For example, Jiang et al*.* [37] proposed a multi-resolution residual connection network for lung tumor volume segmentation and showed that the DSC accuracy of automatic segmentation was 0.74; and Zhang et al*.* [38] improved the Res Net network and applied it to the GTV segmentation of non-small cell lung cancer, and the DSC accuracy of segmentation could reach 0.73. Through these studies, it can be seen that accurate automatic segmentation of the GTV for lung cancer radiation therapy can be achieved using the correct method.

In this study, we proposed a TransResSEUnet2.5D network to explore the accurate segmentation of the GTV for radiation treatment of lung cancer patients. According to the segmentation results, our proposed network segmentation is relatively effective, especially in the margins of the burr, where we automatically segmented the DSC of (84.08 ± 0.04) %. This is due to the special 2.5 D architecture of the TransResSEUnet 2.5D network. 2.5D architecture uses 2D convolutional layers, which can restore edge details in segmentation results more accurately for 2D edge feature information, to extract features in CT images, and it also uses 3D convolutional layers, which extract interlayer information in CT images by using abstract semantic features. Such a special network architecture is compatible with the advantages of both 2D and 3D convolutional layers. Compared with the simplest Unet2D DSC (77.07 ± 0.09) %, 7% higher, which also fully demonstrates the advancement of our research work. The lack of statistically significant differences may be due to the small sample size, but in later experiments the sample size can be expanded this year to explore the statistical significance.

In the test set of automatic segmentation of GTV in 20 lung cancer patients, the DSC metric of the TransResSEUnet2.5D network was higher than all other five network models, and the variance was smaller. It indicates that the automatic segmentation effect of TransResSEUnet2.5D is more stable and the generalization performance of the model is better. HD95 is a measure of the degree of distortion of the segmentation results, and its magnitude is influenced by the number of outlier points [30]. Through statistical analysis, TransResSEUnet2.5D segmented images with greater continuity and produced fewer outliers in 20 test set patients, and the HD95 metric was superior to other network models. Currently, HD95 is in the range of 7.19- 9.35 mm in most studies [39]. In our study, the HD95 of TransResSEUnet2.5D was (8.11 ± 3.43) mm, and it was better than the other models, to some certain, but there was no statistical difference, which might be due to the small sample size. The study by Cui et al*.* [40] used DVNs network to automatically segment lung tumors with DSC of 83.2% and HD95 of 4.57 mm; hence the index of HD95 was superior to our study. One possible reason lies in the differences of the CT thickness between our studies. All patients in our study were treated with IMRT and the layer thickness of their CT was 5 mm, whereas Cui et al*.* [40] studied non-small cell patients treated with SBRT and the layer thickness of their scanned CT was 2 mm or 3.3 mm. Besides, lung cancer patients treated with SBRT had smaller tumors (in the Cui et al*.* [40] study, the GTV mean effective diameter of GTV was 11.039 mm). Nonetheless, the result differences between our studies suggest that there is a need to further improve the segmentation accuracy of our proposed network by regulating the parameters and the depth of iteration during automatic segmentation training. In addition, the amount of data in this study is still relatively small, especially lacking multicenter data, which will affect the robustness of the segmentation model. These are some issues that require further attention in our follow-up research.

The TransResSEUnet 2.5D network proposed by this research achieves the clinical applicability requirements in the indicators of GTV automatic segmentation of DSC and HD95 for lung cancer radiotherapy patients, and also greatly improves the efficiency of radiotherapy delineation. It has been reported that radiotherapy targets for lung cancer are manually delineated by experienced radiotherapy physicians, which took nearly 32 min [41]. Ermiş et al*.* automatically segmented the target area of one glioma patient based on deep learning methods, which took about 10 s [42]. In our study, the automatic segmentation time of GTV for each lung cancer patient was shortened to less than 8 s, about (6.50 ± 1.31) s. Great progress has been made while ensuring accuracy. TransResSEUnet 2.5D network prediction time is longer than Unet 2.5D (p = 0.000), Unet3D (p = 0.000), and ResSEUnet 2.5D (p = 0.001). This might be due to the fact that the TransResSEUnet 2.5D network adds Transformer's modules to the ResSEUnet2.5D, making the model more complex, with more parameters and longer natural prediction times. When the segmentation accuracy is not high, even if the prediction time is short, this is also not clinically meaningful. Therefore, the TransResSEUnet 2.5D network we proposed is of clinical significance.

In summary, on the automatic GTV segmentation task for radiation treatment of lung cancer patients, the TransResSEUnet2.5D network that we proposed can effectively prevent the occurrence of overfitting even when the training set is not large enough, and it effectively mitigates the vanishing gradient problem by repeatedly utilizing the feature maps of different layers during the training process—providing a new method for medical image segmentation.

## Data Availability

The datasets used and/or analysed during the current study available from the corresponding author on reasonable request.

## References

[1] Sung H, Ferlay J, Siegel RL, Laversanne M, Soerjomataram I, Jemal A, Bray F (2021). Global Cancer Statistics 2020: GLOBOCAN Estimates of Incidence and Mortality Worldwide for 36 Cancers in 185 Countries. CA Cancer J Clin.

[2] Siegel RL, Miller KD, Fuchs HE, Jemal A (2021). Cancer Statistics, 2021. CA Cancer J Clin..

[3] Yan T, Guo S, Zhang T, Zhang Z, Liu A, Zhang S, Xu Y, Qi Y, Zhao W, Wang Q, Shi L, Liu L (2021). Ligustilide prevents radiation enteritis by targeting Gch1/BH4/eNOS to improve intestinal Ischemia. Front Pharmacol.

[4] Vinod SK, Jameson MG, Min M, Holloway LC (2016). Uncertainties in volume delineation in radiation oncology: a systematic review and recommendations for future studies. Radiother Oncol.

[5] Byun H, Yu S, Oh J, Bae J, Yoon MS, Lee SH, Chung JH, Kim TH (2021). An assistive role of a machine learning network in diagnosis of middle ear diseases. J Clin Med.

[6] Sumida I, Magome T, Kitamori H, Das IJ, Yamaguchi H, Kizaki H, Aboshi K, Yamashita K, Yamada Y, Seo Y, Isohashi F, Ogawa K (2019). Deep convolutional neural network for reduction of contrast-enhanced region on CT images. J Radiat Res.

[7] Rhee DJ, Jhingran A, Rigaud B, Netherton T, Cardenas CE, Zhang L, Vedam S, Kry S, Brock KK, Shaw W, O'Reilly F, Parkes J, Burger H, Fakie N, Trauernicht C, Simonds H, Court LE (2020). Automatic contouring system for cervical cancer using convolutional neural networks. Med Phys.

[8] Men K, Chen X, Zhang Y, Zhang T, Dai J, Yi J, Li Y (2017). Deep deconvolutional neural network for target segmentation of nasopharyngeal cancer in planning computed tomography images. Front Oncol.

[9] Wang C, Tyagi N, Rimner A, Hu YC, Veeraraghavan H, Li G, Hunt M, Mageras G, Zhang P (2019). Segmenting lung tumors on longitudinal imaging studies via a patient-specific adaptive convolutional neural network. Radiother Oncol..

[10] Zhang F, Wang Q, Li H (2020). Automatic segmentation of the gross target volume in non-small cell lung cancer using a modified version of resnet. Technol Cancer Res Treat.

[11] Çiçek Ö, Abdulkadir A, Lienkamp SS, Brox T, Ronneberger O, Ourselin S, Joskowicz L, Sabuncu M, Unal G, Wells W (2016). 3D U-Net: Learning dense volumetric segmentation from sparse annotation. Medical image computing and computer-assisted intervention – MICCAI 2016. MICCAI 2016.

[12] He K, Zhang X, Ren S, Sun J. Deep residual learning for image recognition[C]//Proceedings of the IEEE conference on computer vision and pattern recognition. 2016: 770–778. https://arxiv.org/pdf/1512.03385.pdf

[13] Glorot X, Bordes A, Bengio Y. Deep Sparse Rectifier Neural Networks[C]// Proceedings of the 14th International Conference on Artificial Intelligence and Statistics (AISTATS). 2011:315–323. https://www.researchgate.net/publication/215616967_Deep_Sparse_Rectifier_Neural_Networks

[14] Maas AL, Hannun AY, Ng AY. Rectifier nonlinearities improve neural network acoustic models[C]//Proc. icml. 2013;30(1): 3. https://ai.stanford.edu/~amaas/papers/relu_hybrid_icml2013_final.pdf

[15] Ioffe S, Szegedy C. Batch normalization: accelerating deep network training by reducing internal covariate shift. In: ICML(2015). https://arxiv.org/abs/1502.03167

[16] Hu J, Shen L, Albanie S, Sun G, Wu E (2020). Squeeze-and-Excitation Networks. IEEE Trans Pattern Anal Mach Intell.

[17] Reifman J, Feldman EE (2002). Multilayer perceptron for nonlinear programming. Comput Oper Res.

[18] Chen Z, Li C, He J, Ye J, Song D, Wang S, Gu L, Qiao Y, de Marleen B, Philippe CC, Stéphane C, Nicolas P, Stefanie S, Yefeng Z, Caroline E (2021). A novel hybrid convolutional neural network for accurate organ segmentation in 3D head and neck CT images. Medical image computing and computer assisted intervention—MICCAI 2021.

[19] Vaswani A, Shazeer N, Parmar N, Uszkoreit J, Jones L, Gomez AN, Kaiser L. Attention is all you need. Advances in neural information processing systems. 2017: 5998–6008. https://proceedings.neurips.cc/paper/2017/file/3f5ee243547dee91fbd053c1c4a845aa-Paper.pdf

[20] Gu J, Bradbury J, Xiong C, Li VOK, Socher R. Non-autoregressive neural machine translation. arXiv preprint arXiv:1711.02281, 2017.

[21] Devlin J, Chang MW, Lee K, Toutanova K. Bert: Pre-training of deep bidirectional transformers for language understanding. 2018. https://arxiv.org/abs/1810.04805

[22] Dosovitskiy A, Beyer L, Kolesnikov A, Weissenborn D, Zhai X, Unterthiner T, Dehghani M, Minderer M, Heigold G, Gelly S, Uszkoreit J, Houlsby N. An image is worth 16x16 words: Transformers for image recognition at scale. international Conference on learning representations. arXiv preprint arXiv:2010.11929, 2020.

[23] Zhou D, Kang B, Jin X, Yang L. Deepvit: Towards deeper vision transformer[J]. https://arxiv.org/abs/2103.11886, 2021.

[24] Liu Z, Lin Y, Cao Y, Hu H, Wei Y, Zhang Z, Lin S, Guo B. Swin transformer: Hierarchical vision transformer using shifted windows[C]//Proceedings of the IEEE/CVF international Conference on Computer Vision. 2021: 10012–10022. https://arxiv.org/abs/2103.14030

[25] Ba JL, Kiros JR, Hinton GE. Layer normalization. https://arxiv.org/abs/1607.06450, 2016.

[26] https://structseg2019.grand-challenge.org/Home/

[27] http://www.csco.org.cn/cn/index.aspx

[28] https://nycancer.com/nccn/

[29] Dice LR (1945). Measures of the amount of ecologic association between species. Ecology.

[30] Van Ginneken B, Heimann T, Styner M. 3D segmentation in the clinic: A grand challenge. MICCAI Workshop on 3D Segmentation in the Clinic: A grand challenge. (2007) https://www.researchgate.net/publication/46688006_3D_Segmentation_in_the_clinic_a_grand_challenge

[31] Li H, Li F, Li J, Zhu Y, Zhang Y, Guo Y, Xu M, Shao Q, Liu X (2020). Comparison of gross target volumes based on four-dimensional CT, positron emission tomography-computed tomography, and magnetic resonance imaging in thoracic esophageal cancer. Cancer Med.

[32] Li S, Xiao J, He L, Peng X, Yuan X (2019). The tumor target segmentation of nasopharyngeal cancer in CT images based on deep learning methods. Technol Cancer Res Treat.

[33] Cardenas CE, Anderson BM, Aristophanous M, Yang J, Rhee DJ, McCarroll RE, Mohamed ASR, Kamal M, Elgohari BA, Elhalawani HM, Fuller CD, Rao A, Garden AS, Court LE (2018). Auto-delineation of oropharyngeal clinical target volumes using 3D convolutional neural networks. Phys Med Biol.

[34] Yang T, Zhou Y, Li L, Zhu C (2020). DCU-net: multi-scale U-net for brain tumor segmentation. J Xray Sci Technol.

[35] Men K, Zhang T, Chen X, Chen B, Tang Y, Wang S, Li Y, Dai J (2018). Fully automatic and robust segmentation of the clinical target volume for radiotherapy of breast cancer using big data and deep learning. Phys Med.

[36] Guo Z, Guo N, Gong K, Zhong S, Li Q (2019). Gross tumor volume segmentation for head and neck cancer radiotherapy using deep dense multi-modality network. Phys Med Biol.

[37] Jiang J, Hu YC, Liu CJ, Halpenny D, Hellmann MD, Deasy JO, Mageras G, Veeraraghavan H (2019). Multiple resolution residually connected feature streams for automatic lung tumor segmentation from CT images. IEEE Trans Med Imaging..

[38] Zhang F, Wang Q, Li H (2020). Automatic segmentation of the gross target volume in non-small cell lung cancer using a modified version of resnet. Technol Cancer Res Treat.

[39] Chen J, Wang KQ, Jian JB, Wang P, Guo ZC, Zhang WX (2022). Research on automatic segmentation of tumor target of lung cancer in CBCT images by multimodal style transfer technology based on deep learning. Chin J Radiat Oncol.

[40] Cui Y, Arimura H, Nakano R, Yoshitake T, Shioyama Y, Yabuuchi H (2021). Automated approach for segmenting gross tumor volumes for lung cancer stereotactic body radiation therapy using CT-based dense V-networks. J Radiat Res.

[41] Vorwerk H, Zink K, Schiller R, Budach V, Böhmer D, Kampfer S, Popp W, Sack H, Engenhart-Cabillic R (2014). Protection of quality and innovation in radiation oncology: the prospective multicenter trial the German Society of Radiation Oncology (DEGRO-QUIRO study) Evaluation of time, attendance of medical staff, and resources during radiotherapy with IMRT. Strahlenther Onkol.

[42] Ermiş E, Jungo A, Poel R, Blatti-Moreno M, Meier R, Knecht U, Aebersold DM, Fix MK, Manser P, Reyes M, Herrmann E (2020). Fully automated brain resection cavity delineation for radiation target volume definition in glioblastoma patients using deep learning. Radiat Oncol.

